# Nanocomposite Cryogel Carriers from 2-Hydroxyethyl Cellulose Network and Cannabidiol-Loaded Polymeric Micelles for Sustained Topical Delivery

**DOI:** 10.3390/polym12051172

**Published:** 2020-05-20

**Authors:** Denitsa Momekova, Ervin Ivanov, Spiro Konstantinov, Filip Ublekov, Petar D. Petrov

**Affiliations:** 1 Department of Pharmaceutical Technology and Biopharmaceutics, Faculty of Pharmacy, Medical University of Sofia, 1000 Sofia, Bulgaria; dmomekova@pharmfac.mu-sofia.bg; 2 Lab for Experimental Chemotherapy, Department of Pharmacology, Pharmacotherapy and Toxicology, Faculty of Pharmacy, Medical University of Sofia, 1000 Sofia, Bulgaria; ervin.ivanov@gmail.com (E.I.); Konstantinov.spiromihaylov@gmail.com (S.K.); 3 PBG GLOBAL LTD., Byalo Pole st. 3, 1618 Sofia, Bulgaria; 4 Institute of Polymers, Bulgarian Academy of Sciences, 1113 Sofia, Bulgaria; fublekov@polymer.bas.bg

**Keywords:** cannabidiol, cryogels, polysaccharides, micelles, drug delivery

## Abstract

In this contribution, we report the development of original nanocomposite cryogels for sustained topical delivery of hydrophobic natural active substances such as cannabidiol (CBD). The cryogels were fabricated by a method involving cryogenic treatment and photo-crosslinking of aqueous systems containing biodegradable 2-hydroxyethyl cellulose (HEC) and CBD-loaded polymeric micelles. The preparation of the water-soluble form of CBD was a key element for the successful drug loading in the one-pot reaction. The main physical, mechanical and biological characteristics of CBD-loaded and blank cryogels such as gel fraction yield, swelling degree, morphology, storage and loss moduli, and cytotoxicity were studied in detail. The advantage of nanocomposite over pure HEC cryogel carriers in terms of achieving a sustained release profile was also demonstrated.

## 1. Introduction

Polysaccharides are abundant low cost natural polymers that can be modified to suit specific needs. Generally, polysaccharides are non-toxic, biocompatible and biodegradable and therefore they have found widespread application as components in food, biomedical, pharmaceutical, and healthcare products [1,2]. Due to environmental concerns, there are increasing efforts from academia and industry to replace the natural polymers for synthetic ones. An attractive field offering great opportunities to utilize polysaccharides is the design and fabrication of advanced drug delivery systems [3,4,5]. In particular, the plenty of functional groups available in polysaccharide macromolecules allows their physical or chemical crosslinking in aqueous media, affording polymer hydrogels. Indeed, a large variety of polysaccharide hydrogels, ranging from nano-sized to macroscopic systems, have been extensively studied as carriers of both hydrophilic and lipophilic bioactive substances. For instance, guar gum succinate—sodium alginate hydrogel beads have been prepared for colon-specific drug delivery [6]. The beads possessed a higher swelling degree at pH 7.4 than at pH 1.2, due to the existence of anionic groups in the polymer chains. The pH-dependent swelling behavior favored the more pronounced release of the model drug, ibuprofen, in pH 7.4 than in pH 1.2. Dinu et al. have prepared chitosan-based hydrogels, containing clinoptilolite, for controlled release of diclofenac sodium and indomethacin [7]. It has been demonstrated that drug delivery preferentially takes place in phosphate buffer saline (pH 7.4) unlike simulated gastric fluid (pH 1.2). Alginate/chitosan hydrogels containing alginate gel-like microspheres have been developed for protein delivery [8]. The encapsulation of bovine serum albumin into microspheres embedded in the hydrogel resulted in a significantly lower release rate than the control hydrogel or microspheres alone. A noteworthy, significant share of hydrogel carriers, especially macroscopic ones, possess a super-macroporous morphology. This is not surprising because the huge pores allow unhindered diffusion of bioactive molecules when the last are released from the matrix. The advantage of super-macroporous hydrogels has been illustrated by Im and Kim [9]. They developed near infrared (NIR)-responsive macroscale delivery systems based on an alginate cryogel with embedded gold nanorods (GNRs) for precisely controlled on-demand release of the chemotherapeutic drug mitoxantrone. Hence, the transduction of NIR light energy to heat was exploited to cause a rapid increase of temperature in a local area, breaking the ionic bonds between polymer and drug molecules. As a result, a quick release of the drug was achieved on-demand.

Recently, we published a paper demonstrating the advantages of nanocomposite macroporous cryogels, based on hydroxypropyl cellulose (HPC) and stabilized polymer core-shell micelles (SPM), for topical delivery of hydrophobic drugs [10]. In that study, nanocomposite cryogels were first prepared by photochemical crosslinking of HPC, in the presence of blank SPM, under cryogenic conditions and then curcumin was loaded in the carriers. The incorporation of hydrophobic micellar domains in the hydrogel matrix was considered crucial for the efficient solubilization and sustained release of curcumin.

Cannabidiol is a non-psychoactive cannabinoid that attracts increasing interest due to its therapeutic potential for treating neuropathic pain, various cancers, multiple sclerosis, inflammation, etc. [11]. However, CBD is a lipophilic compound with low oral adsorption, thus implying relatively low bioavailability when administered orally [12]. It has been suggested that the transdermal delivery of CBD could provide a higher level of blood absorption since the first pass metabolism encountered by an orally administered drug is avoided [13]. Moreover, in a review paper, Grotenhermen concluded that transdermal administration increases the duration of drug action compared with all other routes of application [14]. This is advantageous in users of cannabinoids for control of long-term pain and other applications requiring continuous effects. Paudel et al. evaluated the intranasal and transdermal delivery of CBD with and without permeation enhancers [15]. They found that CBD provides significant plasma drug levels after topical gel application on guinea pigs. Moreover, accounting for the fact that CBD concentration started to decline ~6 h after gel removal, the authors suggested a skin reservoir effect. They attributed this phenomenon to the lipophilic properties of CBD that makes it easier to cross the stratum corneum but makes it difficult to traverse through the aqueous dermis. In order to enhance the transdermal delivery of CBD, systems containing nano-sized carrier such as ethosomes has been developed [16]. 

In this paper, we report the development of an original system for topical sustained delivery of CBD, based on HEC cryogel and nano-sized polymeric micelles. The strategy for fabricating drug delivery systems in this work completely differs from the abovementioned study on HPC/SPM cryogel carriers [10] in the way of drug loading. Here, CBD was first loaded into polymeric core-shell micelles and then the micellar form of the drug was embedded in a cryogel carrier via UV-assisted cryotropic gelation of HEC. This approach allowed in situ loading and direct use of the drug delivery system without any purification procedure. The physico-mechanical properties of cryogels, drug release profile and cytotoxicity potential of the systems were evaluated.

## 2. Materials and Methods

### 2.1. Materials

PEO_101_-*b*-PPO_56_-*b*-PEO_101_ (Poloxamer 407, BASF, Germany), 2-hydroxyethyl cellulose (1.3 KDa, Hercules Inc. Aqualon Division, Wilmington, DE, USA), poly(ethylene glycol) diacrylate (PEGDA, 575 g·mol^−1^, Sigma-Aldrich, Darmstadt, Germany), N,N′-methylenebisacrylamide (BAAm, Sigma-Aldrich, Darmstadt, Germany), H_2_O_2_ (30 vol.% water solution, Labimex Ltd., Sofia, Bulgaria), ethanol (96%, Labimex Ltd., Bulgaria) and cannabidiol (CBD, kindly donated by PBG GLOBAL LTD., Sofia, Bulgaria) were used as received.

### 2.2. Preparation of CBD-Loaded Polymeric Micelles

CBD (10 mg) and copolymer (30 mg) were dissolved in 6 mL of ethanol and then mixed with 2 mL of water. The organic solvent and 1 mL of water were removed from the systems by rotary evaporator under reduced pressure at 40 °C, affording 1 mL aqueous solution of CBD-loaded PM.

### 2.3. Synthesis of Nanocomposite Cryogels

One milliliter of CBD-loaded PM (30 wt.% with respect to HEC) solution was added to 8 mL aqueous solution of HEC (0.1 g) under stirring to obtain a homogeneous mixture. H_2_O_2_ (0.02 mL, 30% wt.% aqueous solution) and BAAm/PEGDA (30 mg; 30 wt.% to HEC), dissolved in 1 mL of distilled water, was added under stirring at room temperature. The mixture was then poured into eight Teflon dishes (2 cm in diameter) forming a 2.5 mm thick layer. Next, the dishes were placed in a freezer at −20 °C for 2 h and the frozen samples were irradiated with full-spectrum UV light for 2 min, using a Dymax 5000-EC UV equipment (400 W metal halide flood lamp) at a dose rate of 5.7 J·cm^−2^·min^−1^. Finally, cryogels were immersed in distilled water (0.5 L) and extracted for one week at room temperature. Water was exchanged four times. The extracted cryogels (an equilibrium water uptake was reached within the period of 7 days) were weighed after blotting their surface by filter paper. Next, the gels were freeze-dried and weighed again. Swelling degree (SD) was determined as follow:

$$SD=\frac{w_{2}}{w_{1}}$$

where *w*_1_ is the weight of the freeze-dried sample, *w*_2_ is the weight of the swollen sample. 

Gel fraction (GF) yield was determined by the equation:

$$GF\;\left[\%\right]=\frac{w_{1}}{w_{0}}\cdot 100$$

where *w*_0_ is the initial weight of HEC, *w*_1_ is the weight of the freeze-dried sample.

The given values for each cryogel are an average of eight samples. The experimental errors of the GF yields and the SD calculations are in the range of 2% to 3%.

### 2.4. Loading of Cannabidoil in Pure HEC Cryogels

A freeze-dried pure HEC cryogel disk (~10 mg; obtained without CBD and PM) was soaked in 1 mL of an ethanol solution of CBD (1 g·L^−1^) and then air-dried at room temperature.

### 2.5. Cannabidiol Release Experiments

The CBD release from pure HEC and nanocomposite HEC/PM cryogels was investigated as a function of time at 35 °C. The dissolution tests were performed in 50 mL acetate buffer (pH 5.5) under constant stirring at 50 rpm. At pre-determined time intervals, 2 mL of the samples were taken from the acceptor phase and the quantity of released CBD was evaluated spectrophotometrically at 235 nm. All samples were measured in triplicate and the mean cumulative percentage of drug release was calculated based on a standard curve of CBD in dissolution medium in the concentration range from 0.002 to 0.1 g·L^−1^ (y = 12.1375x + 0.11934; R^2^ = 0.995).

To determine the total amount of CBD per disk, the disks were placed in 2 mL of distilled water for 10 min and afterward 8 mL of ethanol (96%) were added. The disks were incubated at room temperature for 2 h. After the incubation period, the amount of the extracted CBD was calculated based on a standard curve of CBD in ethanol in the concentration range 0.002 to 0.1 g·L^−1^ (y = 12.13751x − 0.11934, R^2^ = 0.9992).

### 2.6. Cell Lines and Culture Conditions

The cytotoxic activity of CBD either free or loaded in cryogels was assessed against two cell lines: MJ cells (cutaneous T-cell lymphoma, CTCL, Mycosis fungoides) and T-24 cells (urinary bladder cancer). The cell lines were purchased from the German Collection of Microorganisms and Cell Cultures (DSMZ GmbH, Braunschweig, Germany). The biocompatibility of the pure carriers was evaluated on normal mouse fibroblast cells (CCL-1). The growth medium for MJ and T-24 cells was 90% RPMI-1640 + 10% FBS, while the CCL-1 cells were grown in 90 MEM supplemented with 10% horse serum. The cells were grown in a controlled environment—cell culture flasks at 37 °C in an incubator ‘BB 16-Function Line’ Heraeus (Kendro, Hanau, Germany) with a humidified atmosphere and 5% CO_2_.

### 2.7. Cytotoxicity Assessment

The cellular viability after exposure to free CBD or its HEC-based cryogel formulations was evaluated by the MTT-dye reduction assay. The biocompatibility of the carriers was tested on a normal mouse fibroblast (CCL-1) cell line. In brief, exponentially growing cells were plated in 6-well flat-bottomed plates (3 mL/well) at a density of 1 × 10^5^/mL MJ cells (cutaneous T-cell lymphoma, CTCL, Mycosis fungoides) and T-24 cells (urinary bladder cancer) and after 24 h incubation at 37 °C, they were exposed to various concentrations of the tested CBD formulations for 72 h. The CCL-1 cells were plated as described above, but instead of CBD formulations, they were treated with the various concentrations of non-loaded HEC/PM cryogel. A set of at least three wells was used for each concentration. After the contact period, 300 μL of MTT solution (10 g·L^−1^, in PBS) was added to each well. Thereafter, the microplates were incubated for 4 h at 37 °C and the MTT-formazan crystals formed were dissolved through the addition of 3 mL/well 5% formic acid-acidified 2-propanol. The MTT-formazan absorption was recorded using a LabeximLMR-1 microplate reader at 580 nm. Cell survival fractions were calculated as a percentage of the untreated control. In addition, IC_50_ values were derived from the concentration-response curves.

### 2.8. Methods

Dynamic light scattering (DLS) measurements were carried out on a ZetasizerNanoBrook 90Plus Zeta (Brookhaven, NY, USA), equipped with a 35 mW red diode laser, (λ = 640 nm) at a scattering angle of 90°. UV-vis spectra were measured with a UV-1800 Shimadzu spectrophotometer (Shimadzu, Japan). Dynamic rheological measurements of cryogels were performed using a HaakeRheoStress 600 rheometer (Thermo Scientific, Karlsruhe, Germany) with a parallel plate sensor system (20 mm in diameter) and a Peltier temperature controller at 25 °C in controlled deformation (CD) mode. Dynamic storage (G′) and loss (G″) moduli were determined in the 0.3–10 Hz frequency range at constant shear strain, γ = 0.005, which was inside the linear viscoelastic regime. Three runs of each cryogel type were conducted. The cryogels were frozen and freeze dried in an Alpha1-2 Freeze drier (Martin Christ, Osterode am Harz, Germany) at −55 °C and 0.02 mbar for 24 h. The cross-section of cryogels was studied by using a JEOL JSM-5300 SEM (JEOL, Akishima, Japan) scanning electron microscope, operating at 20 kV. Before observations, the gel specimens were fractured, fixed on a glass substrate by nail polish and coated with gold for 60 s. Wide-angle X-ray diffraction (WAXD) scans were obtained on a Bruker D8 Advance ECO diffractometer, operating at 40 kV and 25 mA in Bragg–Brentano geometry with Ni-filtered Cu Kα radiation and a LynxEye-XE detector over the 2θ range of 5–55°, with a scanning rate of 0.02°·s^−1^.

## 3. Results and Discussion

**Figure 1:**
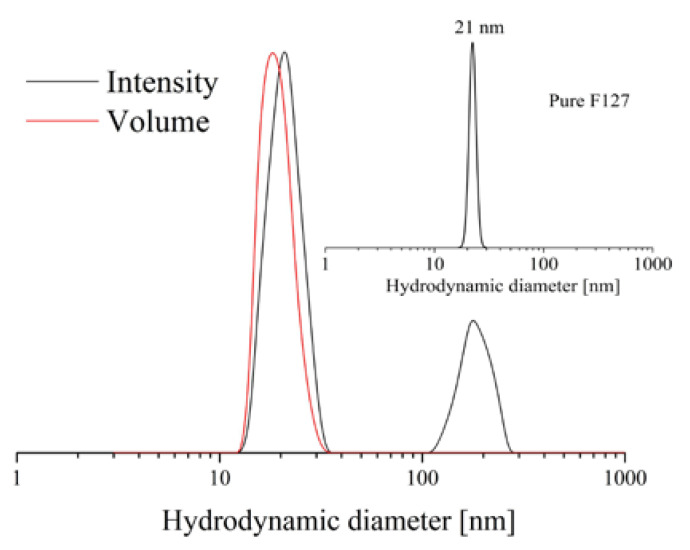
Size distribution of pure (inset) and cannabidiol-loaded polymeric micelles measured at temperature 25 °C and angle 90°. The concentrations of F127 and CBD were 30 and 10 g·L^−1^, respectively.

The method developed for the synthesis of polysaccharide cryogels by photochemical crosslinking in the frozen state requires the use of water as a freezable solvent [17]. The presence of organic solvents such as ethanol, methanol, etc. might hinder both the regular cryo-structuring process and crosslinking reaction [18]. Therefore, the first step of this study involved solubilization of the hydrophobic CBD in aqueous media with the aid of block copolymer micelles. PEO-*b*-PPO-*b*-PEO triblock copolymers are commercially available products (Pluronics, Poloxamers, etc.), well known for their high capacity to solubilize hydrophobic drugs [19]. In particular, PEO_101_-b-PPO_56_-b-PEO_101_ (popular under trade names Poloxamer 407, Pluronic F127, etc.) is available as a pharmaceutical grade product and used for solubilizing active substances in pharmaceutical formulations [20]. For that reason, PEO_101_-b-PPO_56_-b-PEO_101_ (denoted further in the text as F127 for simplicity) was selected to prepare a micellar solution of CBD in water by applying the solvent evaporation technique. First, water was added to an ethanol solution of CBD and block copolymer, followed by evaporation of the organic solvent under reduced pressure. The final concentrations of polymer and CBD were 30 and 10 g·L^−1^, respectively, corresponding to 25% drug loading. DLS measurements of CBD-loaded micelles were conducted to determine the micellar size and size distribution (Figure 1). The intensity-weighted plot revealed the formation of small micelles having a hydrodynamic diameter (D_h_) of 21 nm and relatively narrow dispersity. These results are similar to the characteristics obtained for pure F127 micelles (Figure 1, inset). There is an extra, less intensive peak (D_h_ = 173 nm) in the CBD-containing sample, suggesting the existence of larger aggregates in the colloid solution. However, one can assume that the micelles are the dominant population in the sample as the second peak is not visible in the volume-weighted plot.

**Figure 2:**
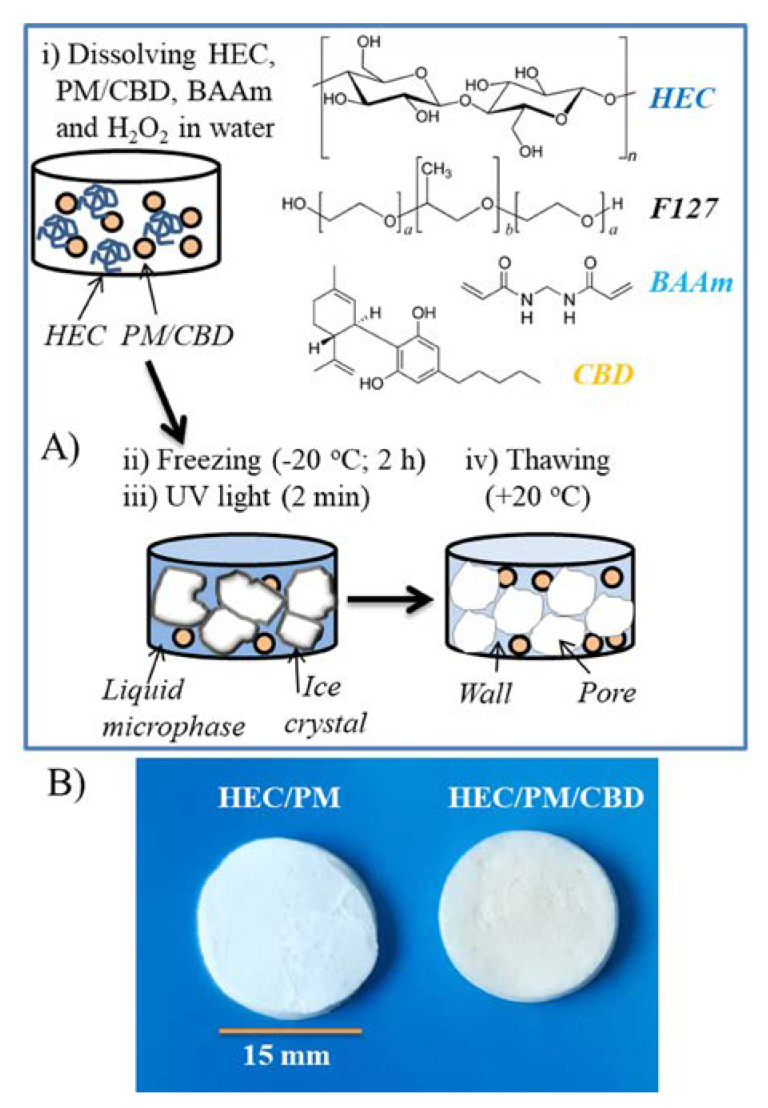
Preparation of nanocomposite cryogels by UV-induced crosslinking in frozen state and thawing (**A**), and digital image of blank and cannabidiol loaded cryogels (**B**).

Next, the micellar solution was mixed with an aqueous solution of HEC, crosslinking agent BAAm (or PEGDA) and photoinitiator H_2_O_2_ (Figure 2A). The mixture was homogenized, frozen and kept at temperature −20 °C for 2 h, and then irradiated with UV-light for 2 min. It has been previously shown that such reaction procedure affords good quality nanocomposite cryogels of high GF yield [10]. The mechanism of formation of nanocomposite super-macroporous cryogel as follows: The cooling of the aqueous mixture at the reported conditions caused the formation of ice crystals (solid phase) surrounded by a liquid microphase (LM). It is known that the solutes, i.e., polymer, initiator, crosslinking agent and micelles are concentrated in the LM, while the crystals are considered a pure form of water [21]. Consequently, the polymer network was formed in the LM by recombination of active (macro) radicals, generated from HEC and crosslinker molecules by the UV-irradiation. In turn, the solid crystals templated the cryogel pores. The thawing of frozen samples afforded spongy materials comprising interconnected pores, filled with free water which can be squeezed by compression. In principle, a polymer network can be obtained by crosslinking of HEC only; however, the involvement of bifunctional molecules (crosslinking agent) into the crosslinking process has been shown to improve the reaction efficacy and mechanical strength of cryogels [22]. In this work, two crosslinking agents, BAAm and PEGDA, were used at the same content (30 wt.% to HEC). The gels obtained with BAAm exhibited slightly higher GF yield than those with PEGDA (Table 1). This is probably due to the existence of more reactive end groups per unit mass, as BAAm has a lower molar mass than PEGDA. The freeze-dried cryogels, containing CBD were a little yellowish unlike the white pure HEC cryogel (Figure 2B). It was found that the presence of CBD in the reaction mixture interferes to some extent the crosslinking process. However, by keeping the drug concentration up to 10% to the HEC mass, one can obtain gels of relatively high yield (80–85%). From a practical point of view the small decrease of GF yield of CBD-loaded cryogels, compared to blank ones, is negligible since the gels were compact, elastic and easy to handle. At a first glance, the swelling behavior of cryogels in water also suggested some difference in the density of polymer network obtained in the presence/absence of CBD (Table 1). Normally, the denser networks swell less in liquids. In the case of cryogels, there are some specific features making the correct interpretation of results slippery. Cryogels are highly porous heterogeneous materials and naturally the water uptake is dominated by the interconnected pores. Typically, the capillary-bound water present in the pores is about 60–70% of the total water. In some cases, this is advantageous because the diffusion of small molecules (drugs, nutrients, etc.) within the gel is facilitated. As a whole, the nanocomposite cryogels uptake a significant amount of water which, in combination with the biocompatibility of polymers, makes them suitable for drug delivery applications.

**Figure 3:**
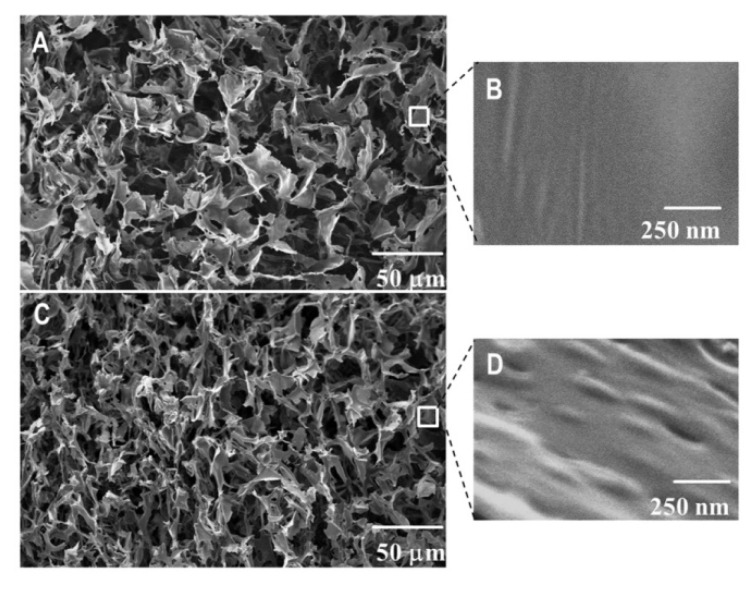
SEM micrographs of pure HEC (**A**,**B**) and drug-loaded nanocomposite HEC/PM/CBD cryogels (**C**,**D**).

The inner morphology of nanocomposite HEC cryogels was studied using scanning electron microscopy. Cryogels were freeze-dried and fractured prior to the analyses. It was anticipated that CBD-loaded micelles are embedded into the HEC matrix due to the cryo-structuring phenomena described above. Therefore, one could expect to see nano-sized micellar structures in the cryogel walls rather than in the pores. For comparison, pure HEC cryogel was analyzed by SEM under the same conditions. Representative micrographs of pure and nanocomposite HEC cryogels are shown in Figure 3. Both materials are super-macroporous and have similar dimensions for the pores (20–30 μm) and wall thickness (1–2 μm). At a high magnification, one can clearly differentiate the smooth surface of pure HEC gel (Figure 3B) and the surface decorated with nano-sized bumps of CBD/PM/CBD containing sample (Figure 3D). Thus, the SEM study confirmed our suggestion that CBD-loaded polymeric micelles were homogeneously embedded in the cryogel walls (polymer matrix).

**Figure 4:**
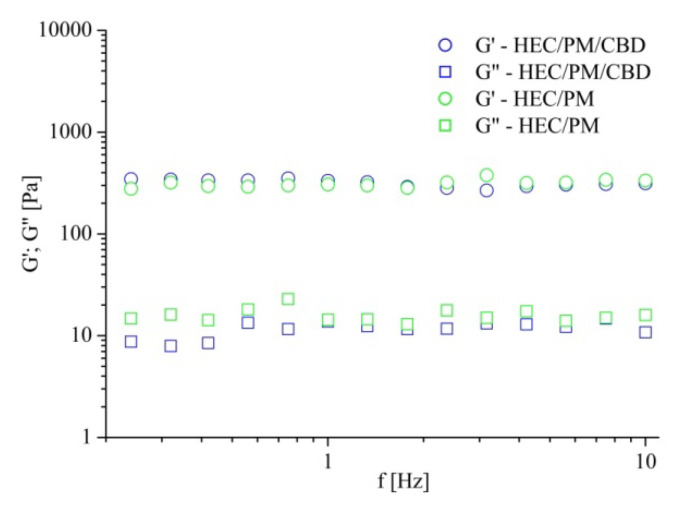
Dynamic rheological measurements of nanocomposite HEC/PM cryogels, prepared with and without cannabidiol, in the 0.3–10 Hz frequency range. BAAm (30 wt.% to HEC) was used as a crosslinking agent.

Nanocomposite cryogels were soft and tear-resistant upon gentle handling. Their viscoelastic characteristics, storage (G′) and loss (G″) moduli were evaluated by dynamic rheological measurements in the oscillation–frequency mode. Figure 4 compares the results from HEC/PM cryogels prepared with and without CBD. In both cases, in the 0.3–10 Hz frequency range, the storage and loss moduli were frequency-independent and G′ was more than one order of magnitude higher than G″. The G′ and G″ values of the two samples were very similar. Definitely, the nanocomposite HEC cryogels exhibited typical properties for chemically crosslinked gels and one may conclude that the incorporation of CBD at the given concentration did not alter the mechanical strength of carrier.

**Figure 5:**
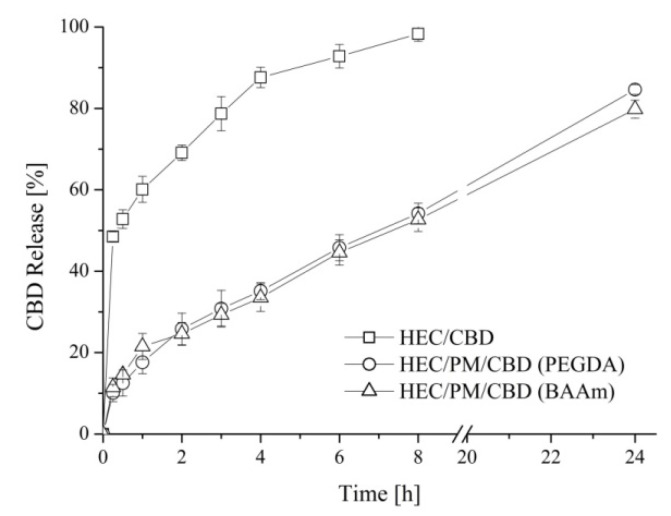
Drug release profiles for cannabidiol-loaded pure HEC and nanocomposite HPC/PM cryogel carriers in acetate buffer (pH = 5.5) at 35 °C. Mean values ± SD (n = 3).

The advantages of the nanocomposite cryogel carrier of CBD, in comparison to pure HEC carrier, were demonstrated by the release experiments. The in vitro tests were performed in acetate buffer of pH 5.5 taking into account that the elaborated cryogels are intended for a potential dermal application and the pH of human skin is about 5.5. The evaluation of CBD release from both pure and nanocomposite HEC cryogels showed a significant difference in the profiles (Figure 5). The pure HEC cryogel carrier was characterized by an initial burst release of CBD. In fact, the carrier released approximately 50% of CBD in the first hour of the dissolution experiment and the process completed within 8 h. 

In the case of nanocomposite HEC/PM cryogels (obtained either with PEGDA or with BAAm), there was no burst effect. These systems were characterized with the pronounced release of CBD for the studied period of 24 h. The observed differences in the release profiles of CDB from pure and nanocomposite cryogel systems are most likely due to the different loading procedure and localization of the active compound, respectively. Indeed, one may assume that by soaking pre-formed HEC cryogel in an ethanol solution of CBD and subsequent solvent evaporation, most of the CBD was entrapped into the gel pores. In the macroscopic pores, the fluid uptake, dissolution and diffusion of CBD are rapid. In contrast, the loading of CBD in micellar cores combined with the cryogenic procedure afforded systems with CBD embedded within the gel walls (polymer matrix). Thus, the release from micelles and diffusion of drug molecules into the polar dissolution medium was decelerated. Noteworthy, the strategy to embed a hydrophobic substance in the cryogel walls via the reported procedure could be accomplished only with a water-soluble form of CBD such as a micellar solution. Besides, the type of crosslinking agents used in the preparation of nanocomposite cryogels did not affect the release behavior of the drug. 

**Figure 6:**
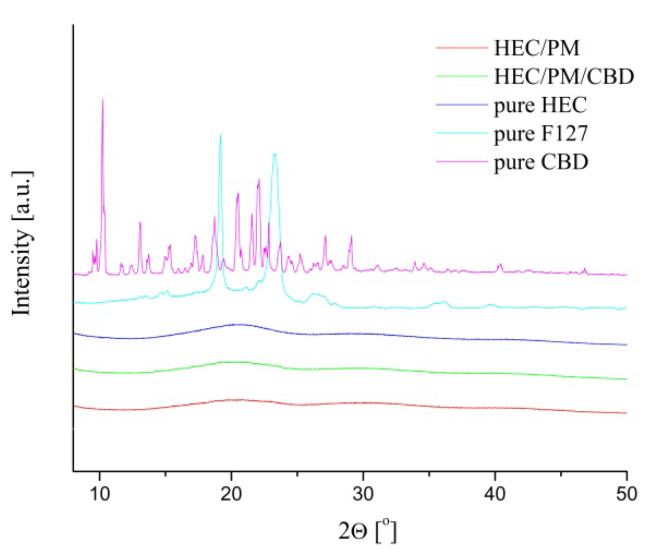
Wide-angle X-ray scattering spectra of pure cannabidiol, F 127 copolymer, HEC cryogel, nanocomposite HEC/PM/CBD, HEC/PM and HEC/CBD samples.

WAXD analysis of freeze-dried samples revealed that CBD, loaded in the nanocomposite cryogel was in an amorphous state (Figure 6). To collect statistical data and to avoid any preferred orientation from the crystals, the measurement was conducted by sample rotation at 10° min^−1^. The patterns of pure CBD and pure F127 showed many diffraction peaks and their positions represent the crystal structure of CBD and F127, respectively. In contrast, in the diffraction patterns of HEC/CBD and HEC/PM/CBD, the crystalline structure of CBD cannot be detected. This phenomenon should be ascribed to the applied preparation procedure, i.e., encapsulation of a small amount of CBD in nano-sized micelles, which was then distributed in the polymeric matrix. The specific microstructure of material obviously did not favor the crystallization of CBD as well as poly(ethylene glycol) segments from the micellar shell. 

The results from WAXD measurements confirmed the amorphization of CBD when the active substance was embedded in the HEC matrix in the form of a micellar solution via cryogenic treatment. This is considered advantageous since the solubilization of amorphous CBD in physiological media is easier. 

**Figure 7:**
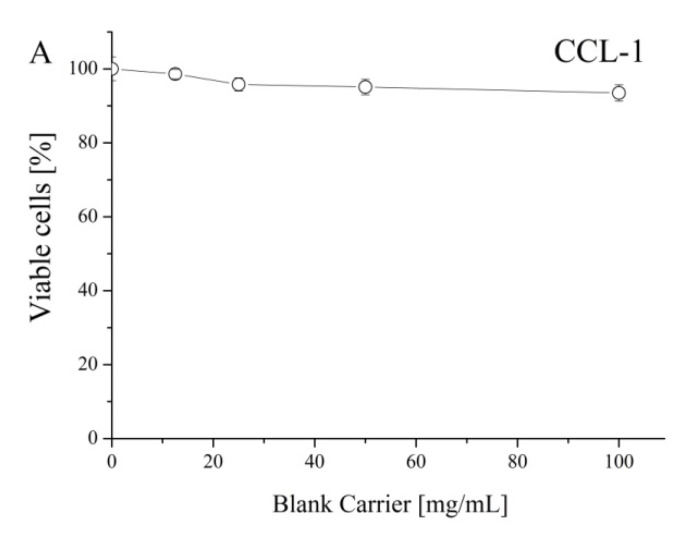

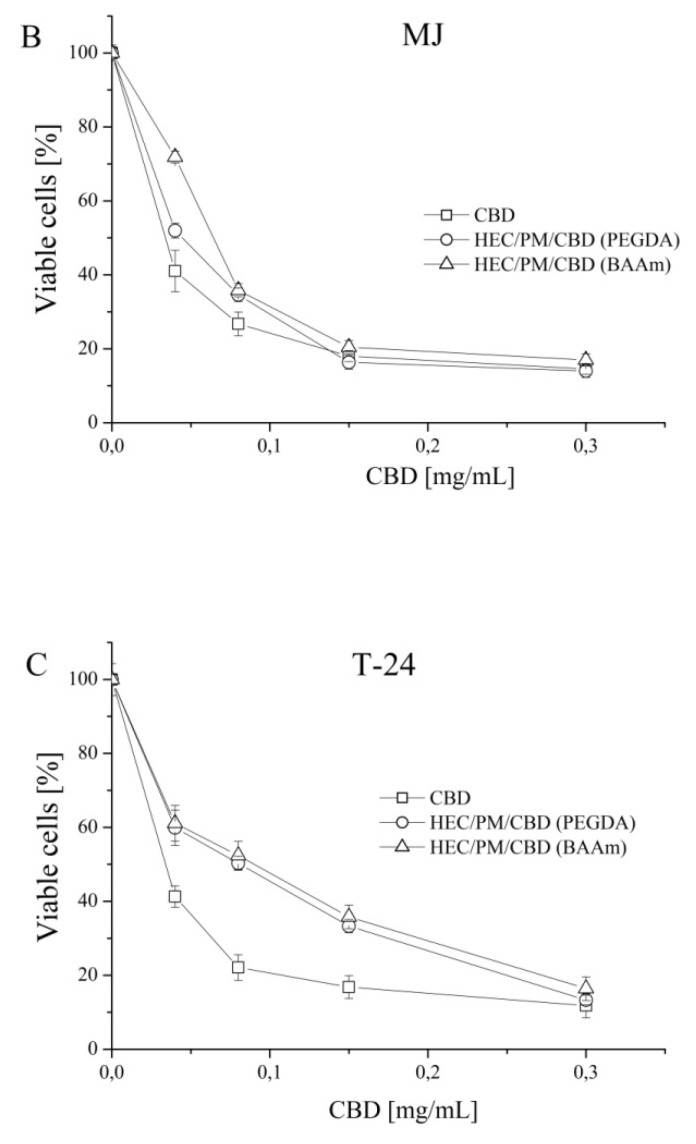
Concentration-response curves determined by the MTT-dye reduction assay after 72 h of continuous exposure for mouse fibroblast cells (CCL-1) and human tumor T-cell lymphocyte (MJ) and urinary bladder carcinoma (T-24) cell lines. Each data point represents an average arithmetic value ± standard deviation of at least 12 independent experiments.

To prove that polymeric carriers do not have intrinsic cytotoxicity, the inhibition effect of blank cryogel systems on the proliferation of mouse fibroblast cells (CCL-1) was studied (Figure 7A). The non-loaded cryogels were devoid of any cytotoxicity since no suppression of the vitality of the treated cells (values in the 94–98% range) was observed. Next, a comparative study regarding the antiproliferative activity of free cannabidiol and its nanocomposite cryogel formulations was carried out. In all experiments, we used a standard cytotoxicity bioassay test, based on the enzymatic reduction of the yellow tetrazolium salt MTT to a violet MTT-formazan by the mitochondrial succinate dehydrogenase in viable cells [23] with slight modifications [24]. The in vitro antineoplastic activity was evaluated against two human tumor cell lines MJ (T-cell lymphocyte) and T-24 (urinary bladder carcinoma). The concentration-response curves are depicted in Figure 7B,C and the equieffective inhibitory concentrations (IC_50_) derived thereof are summarized in Table 2. 

Evidently, both free CBD (applied as ethanol solution) and formulated CBD exhibited concentration dependent cytotoxicity. At lower drug concentrations, the effect of free CBD is more pronounced. The IC_50_ values of the two nanocomposite HEC/PM cryogel systems (synthesized with PEAGA and BAAm) were higher than those obtained for the free drug (with an identical magnitude of modulation) depending on cell type. However, our findings firmly indicate that the tested nanocomposite carriers represent a plausible platform for the effective topical delivery of CBD without compromising its antineoplastic effects in terms of local skin or intravesical application. The in vitro bioassay unambiguously showed that the loaded systems are able to release CBD in a sustained manner and further to retain the tumor inhibitory effects of the natural product in respective in vitro models of mycosis fungoides (CTCL; cell line MJ) and non-invasive urinary bladder cancer (cell line T-24). Those two models represent important neoplastic diseases, whose anatomical localization and biological characteristics allow regional or topical therapy.

## 4. Conclusions

The preparation of a water-soluble form of CBD with the aid of block copolymer micelles allowed in situ loading of the active substance into HEC cryogel carriers in a one-pot reaction. By adjusting the CBD/HEC mass ratio to 1:10, super-macroporous cryogels of relatively high GF yield (80–85%), high water uptake and good mechanical properties were fabricated. Thanks to the preparation method, the CBD-loaded micelles were regularly embedded into the polymer matrix, while the active substance was in an amorphous state. Biological tests revealed that the nanocomposite cryogel carriers preserve the antineoplastic activity of CBD and enable sustained local drug delivery. Such cryogel-based drug delivery systems might be beneficial for patients with cutaneous lesions due to CTCL, as well as for patients with recurrent non-invasive urinary bladder cancer in terms of occlusive skin bandaging and intravesical instillation formulations.

## Figures and Tables

**Table 1:** Gel fraction yield and swelling degree of cryogels obtained by UV-irradiation of frozen aqueous systems containing HEC (1 wt.%), BAAm or PEGDA (0.3 wt.%), PM (0; 0.3 wt.%) and CBD (0; 0.1 wt.%).

Precursors	GF Yield (%)	SD
HEC/BAAm/PM/CBD	84 ± 3	50 ± 2
HEC/PEGDA/PM/CBD	80 ± 2	54 ± 2
HEC/BAAm/PM	90 ± 3	40 ± 1
HEC/PEGDA/PM	87 ± 3	49 ± 2

**Table 2:** Equieffective concentrations (IC_50_) of free and formulated CBD for MJ and T-24 cells.

Sample	IC_50_ (mg·mL^−1^)	
	MJ	T-24
CBD	0.033	0.034
HEC/PM/CBD (PEGDA)	0.046	0.071
HEC/PM/CBD (BAAm)	0.058	0.090
